# Synthesis, Structure, and Function of Human Adenovirus Small Non-Coding RNAs

**DOI:** 10.3390/v12101182

**Published:** 2020-10-19

**Authors:** Tanel Punga, Mahmoud Darweesh, Göran Akusjärvi

**Affiliations:** Department of Medical Biochemistry and Microbiology, Uppsala University, 75123 Uppsala, Sweden; Mahmoud.Darweesh@imbim.uu.se (M.D.); Goran.Akusjarvi@imbim.uu.se (G.A.)

**Keywords:** human adenovirus, sncRNA, VA RNA, mivaRNA, PKR, Dicer, MLP-TSS-sRNA, miRISC

## Abstract

Human adenoviruses (HAdVs) are common pathogens causing a variety of respiratory, ocular and gastrointestinal diseases. To accomplish their efficient replication, HAdVs take an advantage of viral small non-coding RNAs (sncRNAs), which have multiple roles during the virus lifecycle. Three of the best-characterized HAdV sncRNAs; VA RNA, mivaRNA and MLP-TSS-sRNA will be discussed in the present review. Even though VA RNA has been extensively characterized during the last 60 years, this multifunctional molecule continues to surprise us as more of its structural secrets unfold. Likely, the recent developments on mivaRNA and MLP-TSS-sRNA synthesis and function highlight the importance of these sncRNA in virus replication. Collectively, we will summarize the old and new knowledge about these three viral sncRNAs with focus on their synthesis, structure and functions.

## 1. Human Adenoviruses

Human adenoviruses (HAdVs) are common pathogens causing a variety of gastrointestinal, respiratory, and ocular diseases in humans [1,2]. In addition to their pathogenicity, HAdVs have gained a lot of attention as the experimental tools to study various molecular biology mechanisms and as the robust therapeutic tools suitable for disease treatment and prevention. Extensive cell biology and biochemistry studies using HAdV infections or HAdV-encoded proteins/RNAs have revealed the molecular mechanisms involved in anti-viral immune responses, virus uptake, mRNA processing, DNA replication and protein degradation [3]. This basic molecular virology knowledge has been instrumental to design genetically modified adenoviruses for therapeutic purposes. Indeed, genetically modified HAdVs have been successfully used for cancer treatment since they can specifically induce tumor cells lysis [4]. Modified adenoviruses can also be used as the delivery vehicles for vaccination purposes. The recent promising studies using genetically modified human and simian adenoviruses as the vectors to vaccinate against SARS-CoV2 infections clearly underscore the importance of adenoviruses in population-wide clinical applications [5,6].

More than 100 different HAdV types have been described so far (http://hadvwg.gmu.edu). However, most of the functional studies have been carried out using the genetically almost identical virus types 2 and 5 (HAdV-2 and HAdV-5) [7]. HAdVs can infect a variety of cell types and they typically establish a lytic infection causing fast and efficient recipient cell lysis [3]. In addition to the relatively well-characterized lytic infection, some virus types, such as HAdV-2 and HAdV-5, can also establish enigmatic long-term persistent infections in B and T cells [8,9].

To accomplish the lytic or persistent modes of replication, HAdVs have to reprogram both the host cell and the viral gene expression machineries [10]. For that purpose, HAdV encode small non-coding RNAs, which can control the efficiency of virus replication [11,12,13].

## 2. HAdV and Small Non-Coding RNA 

By definition, a non-coding RNA (ncRNA) is an RNA molecule that is not translated to a protein. The ncRNAs can be grouped based on their nucleotide length, function, regulatory potential and subcellular localization. The most common grouping takes into consideration the nucleotide chain length of the ncRNAs. Hence, a ncRNA shorter than 200 nucleotides is regarded as a small non-coding RNA (sncRNA). This includes small interfering RNA (siRNA), microRNA (miRNA) and piwi-interacting RNA (piRNA). Further division into small (18–31 nucleotides) and medium (31–200 nucleotides) sncRNAs can be applied to this group. In contrast, a ncRNA longer than 200 nucleotides is considered as a long non-coding RNA (lncRNA) [14].

**Figure 1:**
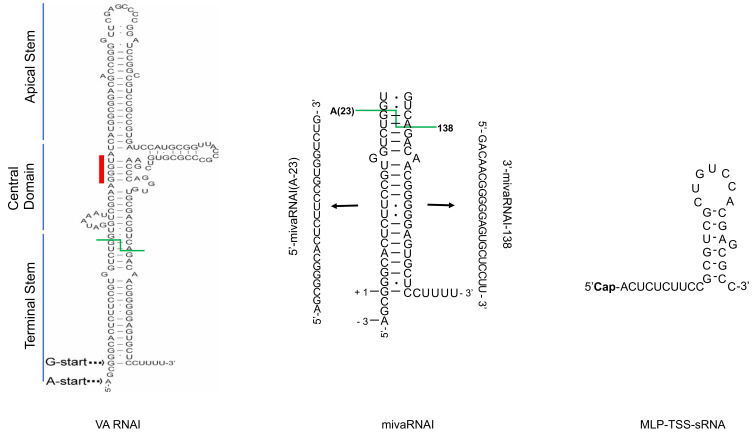
Structural overview of three Human adenovirus (HAdV) small non-coding RNAs (sncRNAs): virus-associated RNAI (VA RNAI), mivaRNAI and MLP-TSS-sRNA. The VA RNAI and mivaRNAI sequences are derived from HAdV-5 [22]. The MLP-TSS-sRNA sequence is from HAdV-37 [16]. Abbreviations: Cap; (m7G)-cap structure. Red line indicates the conserved tetranucleotide sequence (GGGU-ACCC), green line shows Dicer cleavage site within the terminal stem.

Multiple studies have revealed that significant changes in RNA accumulation occur both within the host cell and virus transcriptomes in HAdV-infected cells [10]. In addition, high-throughput RNA deep sequencing experiments have identified a large number of novel HAdV transcripts, that escaped detection by classical biochemistry methods, such as Northern blotting and RT-PCR [15,16,17]. Historically, the short-read RNA sequencing technologies have been used to identify novel HAdV transcripts [10]. A number of studies have demonstrated the existence of multiple viral sncRNAs, with a size length of 20–35 nucleotides, in HAdV-infected cells [16,17,18,19,20,21,22]. However, only a few of these viral sncRNAs have been characterized regarding their synthesis, structure and function. In the present review we will discuss the recent developments on how the HAdV encoded sncRNAs, VA RNA, mivaRNA and MLP-TSS-sRNA (Figure 1), regulate the virus lifecycle.

## 3. VA RNA 

Undoubtedly, the best characterized and studied HAdV sncRNA is the virus-associated RNA (VA RNA) (Figure 1), which was identified more than 60 years ago and is considered as the first viral sncRNA ever described [23].

### 3.1. Synthesis

VA-RNA is a remarkably abundant, medium-sized sncRNA, transcribed from the HAdV genome during the late phase of infection. Most of the HAdV types contain two VA RNA genes: VA RNAI and VA RNAII. In the text, we use VA RNA to describe both VA RNAI and VA RNAII, whereas if a particular feature applies to a specific VA RNA, we indicate it. VA RNAI is the major species with an expression level of >1 × 10^8^ copies/cell at late times of infection, whereas VA RNAII, which is not present in all the HAdV types, is the minor species expressed at around 5 × 10^6^ copies/cell [24,25,26]. Both genes are positioned immediately upstream of the L1 region in the major late transcription unit [26]. The VA RNA genes are arranged in a head-to-tail fashion, separated from each other by a 98-nucleotide spacer sequence in the case of HAdV-2 [27,28]. Why most HAdV types have two VA RNA genes is unclear, although it has been speculated that the VA RNAII gene might be the result of a VA RNAI gene duplication [28]. Interestingly, the VA RNAII gene is prone to accumulate mutations [29,30]. In a recent HAdV-7 outbreak in Wuhan, China, three thymine deletions were found in the VA RNAII transcriptional termination element [29]. This deletion increased expression of the adjacent virus gene, L1 52/55K, and promoted virus growth, indicating that VA RNAII mutations may associate with severe HAdV-7 infections [29]. The VA RNA genes in most HAdV types are about 160 nucleotides long with a slight variation in length due to a small heterogeneity at both the 5’ and 3’ ends of the molecules [24].

A peculiar feature of the VA RNA genes is that they are transcribed by cellular RNA polymerase III (RNAPIII). Since cellular tRNA genes are also transcribed by the RNAPIII, it has been hypothesized that the VA RNA genes may have evolved from a cellular tRNA gene [31]. Transcriptional regulation of both VA RNA genes is similar. Two highly conserved intragenic promoter elements, known as box A and box B, are needed for VA RNAI transcription since their deletions or mutations in these boxes reduces VA RNAI transcription [31,32]. Notably, the HAdV-2 VA RNAI transcription unit contains two transcription initiation sites. The major G start is responsible for about 75% of the total VA RNAI [33], whereas the minor A start site, located three nucleotides upstream of the G start site, accounts for the rest of VA RNAI molecules (Figure 1) [34,35,36]. Usage of different VA RNA transcription start sites seems to be a common feature among different HAdV types. Indeed, high-throughput RNA sequencing experiments have shown that HAdV-4, HAdV-5, HAdV-11 VA RNAI and HAdV-4, HAdV-37 VA RNAII transcripts start with both G and A nucleotides, denoting the usage of aforementioned G and A transcription start sites, respectively [22]. Since VA RNAs are transcribed by the RNAPIII, the 3’-ends of the transcripts are generated by termination of transcription at DNA terminator element; a stretch of four or more dT residues on the non-template DNA strand [37,38]. The HAdV-2 VA RNAI gene contains also a second termination element, about 40 nucleotides downstream of the main termination signal [39]. Potential RNAPIII read-through and usage of the second termination element may explain occasional detection of longer, ca. 200 nucleotides VA RNAI species in the HAdV-2 infected cells [40,41]. Remarkably, even longer, 690 and 950 nucleotide VA RNA containing transcripts, have been detected in the HAdV-2 infected cell nuclei [27]. It remains to be tested whether other HAdV types also encode for longer VA RNA species and what function, if any, these long VA RNAs serve in virus-infected cells. Here, the long-read RNA deep sequencing methods should make it possible to characterize the complexity of VA RNA-containing transcripts [15].

The HAdV-2 VA RNAs are made in approximately equal amounts during the early phase of virus infection [25]. The VA RNA accumulation pattern changes drastically after the initiation of virus DNA replication, as it increases the amount of DNA templates for RNAPIII. Both VA RNA genes compete for the same RNAPIII machinery. However, the VA RNAI promoter is stronger compared to the VA RNAII promoter. This results in a drastic increase in the accumulation of VA RNAI during the late phase of infection [25]. Notably, lack of VA RNAI expression enhances VA RNAII transcription during the late phase of infection, corroborating the idea that transcriptional competition controls the accumulation of the two VA RNA species [42].

### 3.2. Structure

The primary nucleotide sequence of VA RNAI and VA RNAII varies significantly between virus types (e.g., HAdV-5 versus HAdV-37) but is well conserved between members of the same species (e.g., HAdV-2 versus HAdV-5). Sequence alignments have revealed that there are three main VA RNA sequence elements conserved between different HAdV types [24,43]. These elements are box A and box B, present in the terminal and apical stem, respectively, essential for RNAPIII transcription, a complementary tetranucleotide sequence GGGU-ACCC, present in the central domain of the molecule and the RNAPIII terminator sequence of four uridine nucleotides at the 3’ end of the molecule (Figure 1) [24,43]. The secondary structure of HAdV-2 VA RNAI has been intensively studied using different biochemical and computational methods [12,43,44,45,46,47,48,49,50]. Since the VA RNA binds to the cellular anti-viral protein kinase PKR, most of the structural studies describe VA RNA structure in relation to its binding and inactivation of the PKR protein (see Section 3.3.1.). Both VA RNAI and VA RNAII form a hairpin-loop structures with three conserved structural domains: an apical stem, a central domain and a terminal stem structure (Figure 1) [11,12,43,44].

The elongated apical stem folds into a very stable and strong hairpin structure. This exceptionally thermostable domain is resistant to denaturing agents (e.g., 6M urea), which obstruct the electrophoretic mobility of VA RNAI [44,51]. Since the apical stem is sensitive to ssRNA- and dsRNA-specific RNases, it has been proposed that this domain exists as two functionally non-equivalent structures with different binding and inhibitory activities towards the PKR protein [43,52]. Even though the significance of these structures has remained enigmatic, it is accepted that the apical stem is needed for the VA RNAI physical interaction with the PKR protein [47,48,51,52,53,54,55].

VA RNA central domain is the most complex structure linking together the apical and terminal domains. The central domain contains the conserved tetranucleotide GGGU-ACCC stem and a pseudoknot structure, which both contribute to the tertiary structure of the central domain (Figure 1) [46,48,49,50]. Mutations within the central domain do not affect VA RNAI binding to PKR, but instead alter the structural conformation of VA RNAI, which prevents it from inactivating PKR [45]. Based on the chemical probing and small angle x-ray scattering measurements the apical stem and central domain form an extended duplex that binds a single PKR monomer with high affinity, in that way inhibiting activation of PKR [48]. A recent 2.7 Å crystal structure of the apical and central domains revealed that VA RNAI is sharply bent into a “V” shape [46]. Based on this study, the coaxially stacked tetranucleotide stem and the apical stem are necessary for PKR inhibition. Surprisingly, the central domain pseudoknot resembles codon-anticodon interaction and is crucial for the capacity of VA RNAI to block PKR activation [46]. The exact mechanism how the central domain blocks PKR activation is still unclear. It has been proposed that the central domain may prevent PKR dimerization [45,46,48]. Alternatively, a direct contact of the central domain with the PKR kinase domain may block PKR enzymatic activity [46,53]. Since there is no crystal structure of the VA RNAI-PKR complex available, future high-resolution structural studies are needed to reveal the mechanistic details of the VA RNAI-PKR interaction(s).

The terminal stem may stabilize the central domain, but it is not as stable as the apical stem [44]. The terminal stem contains the box A element for VA RNA transcription. Since mutations in box A can have a detrimental effect on VA RNA transcription by RNAPIII [31,32], this part of the terminal stem is highly conserved [24,43]. Even though the terminal stem binds to PKR under some experimental conditions, it is not needed to inhibit PKR activity [47]. Rather, the terminal stem is a substrate for the Dicer endoribonuclease, which generates virus-specific miRNAs, so-called mivaRNAs (see Section 4). Further, the terminal stem structure can control activity of the Dicer enzyme, optimal binding of the OAS1 enzyme as well as the nuclear export of the VA RNA molecules (see Section 3.3.1. and Section 3.3.5.) [11,12].

### 3.3. Function

**Figure 2:**
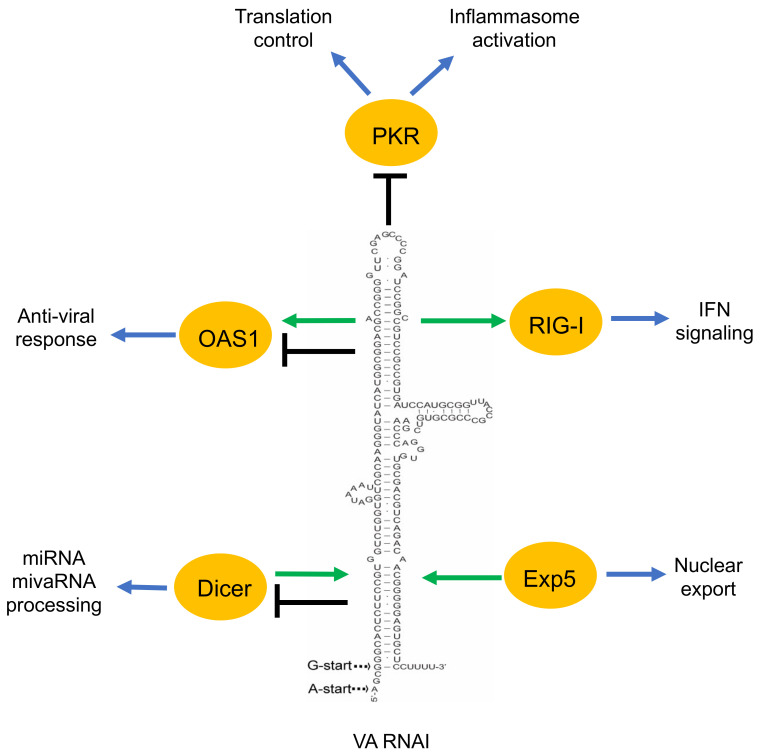
A simplified overview of the VA RNAI functions described in the text. Green arrow indicates the positive effect of VA RNAI to its interacting proteins (OAS1, RIG-I). Moreover, the positive effect of the proteins (Dicer, Exp5) on VA RNAI is shown by a green arrow. Black terminated line indicates the negative effect of VA RNAI on its associating proteins. VA RNAI has a dual effect on some of the proteins (OAS1, Dicer).

The HAdV-5 mutant, dl331, which is defective in VA RNAI expression has been essential for the dissection of the function of the VA RNAs [56]. This mutant virus has severely reduced viral late protein synthesis and virus yield, supporting the conclusion that VA RNAI plays a critical role during virus growth [56]. Elimination of VA RNAI expression reduced virus growth about 20-fold while a deletion of VA RNAII had essentially no impact on virus multiplication. However, a VA RNAI/VA RNAII double mutant showed an approximately 60-fold reduction in virus growth, suggesting that VA RNAII somehow supports virus growth by a so far unknown mechanism [42]. In conclusion, VA RNA, and particularly VA RNAI, functions as an efficient pro-viral factor needed for virus growth. Surprisingly, this medium-sized sncRNAs interfere with multiple cellular processes, which will be reviewed below (Figure 2).

#### 3.3.1. VA RNAI and PKR

VA RNAI interference with the interferon inducible double-stranded RNA (dsRNA)-activated protein kinase (PKR), can explain the essential role of VA RNAI in virus-infected cells [56,57]. PKR is a serine/threonine protein kinase, which plays an important role in mRNA translation, regulation of apoptosis, inflammation, and cell proliferation [58]. An astonishing feature of PKR is that it can sense cellular stress signals and react to them accordingly. The key here is that the PKR protein binds to and gets activated by double-stranded RNA species (dsRNA). Activation of PKR by dsRNA is length-dependent and requires at least 30 base pairs on the activator dsRNA. Mechanistically, the dsRNA binding causes PKR dimerization and autophosphorylation, which are needed for its function as a protein kinase [59]. Activated PKR phosphorylates Serine 51 in the α-subunit of the eukaryotic translation initiation factor 2 (eIF2), causing a stable association of phospho-eIF2 with the GTP recycling factor, eIF2B. Since eIF2B is the limiting factor in translation initiation, this sequestering results in a failure of eIF2B to recycle eIF2-GDP to its active from eIF2-GTP, which is needed for ribosomal 43S pre-initiation complex association with the mRNA [60]. The consequence will be a global inhibition of cap-dependent protein synthesis in PKR activated cells. 

The anti-viral activity of PKR relies on the recognition of dsRNA generated during a virus infection, which results in the signaling cascade described above that blocks virus amplification. In the case of HAdV, it is believed that the activator dsRNA is produced by the bidirectional transcription of the two viral DNA strands that occurs at a high rate in the late-virus infected cells [61]. Such nuclear precursor RNAs have the potential to form dsRNA duplexes and function as the activators of PKR. Many viruses encode protein or non-coding RNA antagonists to counteract the anti-viral functions of PKR [62]. In the case of HAdV, VA RNAI serves this function as it can directly interact with the PKR protein. The two dsRNA-binding domains in PKR bind to the apical stem of VA RNAI, whereas the central domain contributes structural features that makes VA RNAI an inhibitor of PKR activation [12,46,54,55,63]. Since PKR binding to VA RNAI or dsRNA appears to be mutually exclusive [64], the simplest explanation would be that VA RNAI, which is produced in humongous quantities (10^8^ copies/cell), efficiently outcompetes activator dsRNA for binding to PKR. However, this competitive binding model raises a question why VA RNAI behaves as an inhibitor instead of being an activator since VA RNA essentially is a long dsRNA molecule (Figure 1). One possible explanation here is that VA RNAI is over-represented with wobble base pairs, which may convert it to a pseudo-activator [46] that due to its structural constraints binds to but does not activate PKR. Notably, disruption of the essential central domain pseudoknot structure converts VA RNAI from an inhibitor into an efficient PKR activator [45]. Taken together, it is likely that a specific structural conformation of the VA RNAI-PKR complex rather than physical sequestering of PKR by VA RNAI is needed for PKR inhibition [12,45,46,65].

#### 3.3.2. VA RNAI and 2’-5’ Oligoadenylate Synthetase

Another well-known dsRNA sensor, able to recognize virus infections and induces an anti-viral response in virus-infected cells, is 2’-5’ Oligoadenylate Synthetase (OAS) [66]. In the presence of dsRNA, the OAS enzyme polymerizes ATP into a 2’-5’-oligoadenylate, which activates the latent form of ribonuclease L (RNaseL). The active form of RNaseL in turn degrades both cellular and viral RNA thereby inhibiting virus growth [67]. The human genome encodes a family of three catalytically active OAS enzymes (OAS1, OAS2, and OAS3), which have different sensitivities to dsRNA dependent on its length and sequence [66]. Since the OAS proteins show anti-viral activities, many viruses encode antagonists against the OAS proteins [66].

Analogously to PKR, the OAS1 protein binds to VA RNAI, although the binding efficiency is low, about 10% compared to a perfectly base-paired dsRNA of similar size [68]. However, in contrast to PKR, VA RNAI binding does not inhibit OAS1 but rather activates it and thereby enhances 2’-5’-oligomer synthesis [68]. Mutational studies by Conn and colleagues have shown that the optimal OAS1 activation in vitro requires the 3′-end pyrimidine-rich (CUUU or UCCC) single-stranded sequence on VA RNAI [69]. Deletion of the 3’ single stranded CUUU or replacement of it with a purine-rich sequence (GAAA or AGGG) abrogated the OAS1 activation by VA RNAI. Since RNAPIII termination leaves VA RNAI with 3’ single-stranded UUUU sequence, it is possible that VA RNAI activates OAS1 via this particular short sequence element also in vivo. Activation of OAS1 by VA RNAI seems to be counterproductive for virus growth since it may enhance RNaseL-mediated viral RNA degradation. One potential explanation how HAdV may escape from OAS1 activation is a specific VA RNAI processing by the cellular endoribonuclease Dicer. Interestingly, a non-natural truncated VA RNAI, which lacks the entire terminal stem (TSΔ21), has a higher affinity for OAS1 compared to the full-length VA RNAI and may behave as a pseudo-inhibitor of OAS1 [70]. This kind of shorter VA RNA structure will be generated as a byproduct when Dicer cleaves off almost the whole terminal stem of VA RNA, generating only apical stem and central domain (AS-CD) containing VA RNAI molecules (Figure 1) [19,22]. Notably, this Dicer cleaved VA RNAI(AS-CD) fails to activate OAS1, and thus it may act as the pseudo-inhibitor of OAS1 activation [12].

#### 3.3.3. VA RNAI Interference with Type I Interferon

The cellular innate immune response plays a critical role for the host to sense a virus infection. Notably, virus infections produce virus-derived short dsRNA or 5’-triphosphorylated RNAs that are recognized by cytosolic pattern recognition receptors like the retinoic acid-inducible gene I (RIG-I) and melanoma differentiation-associated gene 5 (MDA-5). Activation of RIG-I or MDA-5 triggers, via a complex signaling cascade, the phosphorylation and nuclear translocation of the interferon regulatory factor 3 (IRF-3) and the nuclear factor κB (NF-κB). Activated IRF-3 and NF-κB enhance the production of inflammatory cytokines and type I interferon [71].

Since the VA RNAs are transcribed by RNAPIII, their first 5’-end nucleotide is triphosphorylated [34], which triggers RIG-I downstream signaling [72]. Truly, VA RNAI and VA RNAII interact with RIG-I, which coincides with an increased type I interferon (IFN) synthesis in a RIG-I-dependent manner [73,74]. Further, infection of mouse embryonic fibroblasts (MEFs), and granulocyte-macrophage colony-stimulating factor generated bone marrow-derived dendritic cells (GM-DCs), with VA-RNA deletion mutant sub720 showed a reduced IFN-β accumulation compared to a wild-type virus infection [74]. Transient overexpression of the VA RNA also increased IFN-β accumulation in MEFs, suggesting that VA RNA can target IFN-β signaling independently of the virus infection. Therefore, since VA RNAs exhibit immune-stimulatory effects, care must be taken when designing HAdV-based viral vectors for therapeutic and prophylactic (i.e., vaccination) applications [74].

#### 3.3.4. VA RNAI and the Inflammasome

Immune cells can rapidly sense a viral infection via a group of proteins that upon sensing viral associated molecular patterns (PAMPs) are able to form a cytosolic multiprotein inflammasome complex. These complexes in turn promote a proteolytic cleavage and secretion of pro-inflammatory cytokines like interleukin 1β (IL-1β) and interleukin 18 (IL-18) [75]. In addition to these cytokines, the activated inflammasome also causes extracellular release of the HMGB1 protein, which amplifies the inflammatory process [76]. PKR appears to physically interact with several key inflammasome components, including NLRP3 (NOD-like receptor family pyrin domain-containing 3) [76], and ASC (Apoptosis-associated speck-like protein containing a caspase recruitment domain) [63]. Since VA RNAI binds to and inhibits PKR activation, it was hypothesized that VA RNAI may also block the inflammasome [63]. Indeed, a HAdV-5 infection of the PMA differentiated macrophage-like THP-1 cell line resulted in an inhibition of the NLRP3 inflammasome. In contrast, infection with an HAdV lacking VA RNAI expression failed to inhibit the NLRP3 inflammasome. Conversely, a synthetic VA RNAI molecule was able to inhibit the NLRP3 inflammasome activation in the absence of an HAdV infection.

Mechanistically, the capacity of VA RNAI to block PKR activation seems to be caused by the inhibitory effect of VA RNAI on PKR activity since the disruption of base-pairing within the conserved tetranucleotide GGGU-ACCC stem blocked VA RNAI as an inhibitor of inflammasome activation [63]. Interestingly, in the same work, VA RNAI was found to inhibit the tyrosine Y146 phosphorylation of ASC, which has been proven to be essential for inflammasome activation [77]. PKR is a characterized serine threonine kinase. However, PKR also has been shown to autophosphorylate tyrosine residues in the kinase domain during the activation process [78]. This finding opens up the possibility that VA RNAI may inhibit inflammasome activation via an inhibition of a potential tyrosine kinase activity of PKR. Since VA RNAI blocks NLRP3 inflammasome activation, this knowledge may help to design sncRNA-based therapeutics against overactivation of the inflammasome [63].

#### 3.3.5. VA RNAI and miRNA Biogenesis

Strikingly, VA RNA folds into a stem-loop structure which resembles cellular pre-miRNAs (Figure 1). This structural feature and the fact that other DNA viruses, such as herpesviruses encode viral miRNAs, urged different research groups to study whether the VA RNAs were processed by the miRNA machinery [18,79].

One of the first indications that the VA RNAs might be involved in the miRNA biogenesis pathway came from the studies showing that, similar to pre-miRNA export [80,81], the nuclear export of VA RNAI needs the nuclear export protein Exportin 5 (Exp5) [82,83]. Mutational analysis showed that the VA RNAI terminal stem and its single-stranded 3´end are important for the interaction with the Exp5 protein [83]. Thus, VA RNAI seems to compete with pre-miRNAs for interaction with Exp5 [84]. In addition, VA RNAI expression may also reduce cytoplasmic accumulation of the Dicer protein since export of this particular mRNA seems to need the Exp5 pathway as suggested by Bennasser and co-workers [85]. Based on their experiments, transient expression of VA RNAI inhibits Dicer mRNA-Exp5 interaction, thereby diminishing Dicer mRNA export. Collectively, VA RNA will intrude with the Exp5-driven nuclear export pathway to specifically reduce pre-miRNA as well as Dicer mRNA export to the cytoplasm, something that will promote HAdV growth.

A particular feature of the VA RNA is that they accumulate to extremely high levels in the virus-infected cells. Because of their structural resemblance to pre-miRNAs, VA RNA function as the RNA decoy molecules for the Dicer protein [18,84]. Both VA RNAI and VA RNAII can function as competitive substrates to diminish Dicer endonuclease activity on pre-miRNA substrates [18]. In another study, VA RNAI was shown to form a specific complex with Dicer, which resulted in an efficient inhibition of pre-miR30 cleavage [84]. Thus, VA RNA seems to target different nodes in miRNA biogenesis; pre-miRNA export, Dicer accumulation and its activity, to control efficient virus replication.

## 4. mivaRNA 

As mentioned above, VA RNAs interfere with the miRNA biogenesis components, particularly with the Dicer protein. In fact, both VA RNAI and VA RNAII can be cleaved into cellular miRNA-like molecules, so-called mivaRNAI and mivaRNAII, respectively (Figure 1) [18,20,21,35,86,87,88].

### 4.1. Synthesis and Structure

Essential for mivaRNA biogenesis is the cellular endoribonuclease Dicer, which specifically cleaves within the terminal stem of VA RNAI and VA RNAII. This cleavage causes a predominant accumulation of ca. 21–23 nucleotide long mivaRNA species, which size-wise resemble mature cellular miRNA [18,20,21,86,87,88,89]. MivaRNAs are abundant, comprising up to 47% of the total miRNA pool in the HAdV-5-infected cells [21]. High-throughput small RNA sequencing experiments have further confirmed that the VA RNA terminal stem is the universal substrate for Dicer. At least four different virus types, HAdV-4, HAdV-5, HAdV-11, and HAdV-37, produced mivaRNAs in the infected cells [22]. Interestingly, VA RNAII seems to be a more favored Dicer substrate despite of the fact that it is expressed at only 5% of VA RNAI in infected cells when compared to VA RNAI [21,88]. Depletion of the Dicer enzyme with siRNA in virus-infected cells reduced mivaRNA accumulation, thus validating that the mivaRNAs are indeed Dicer cleavage products [22,86]. A closer inspection of the mivaRNA 5’- and 3’-ends revealed that Dicer has alternative cleavage sites in the terminal stem, thus generating heterogeneous 5′- and 3′-mivaRNA species in the infected cells [22]. The exact Dicer processing site is important as variations in the cleavage site generates mivaRNAs with different seed sequences that, therefore, may target different mRNAs. For example, HAdV-5 VA RNAII is cleaved at two locations by Dicer. One cleavage generates a minor (23 nucleotide) 5′-mivaRNAII(G-23) and a major 3′-mivaRNAII-136, whereas the second cleavage site generates a major (21 nucleotide) 5′-mivaRNAII(G-21) and a minor 3′-mivaRNAII-138. In contrast, Dicer cleaves only at one location in HAdV-5 VA RNAI generating both the major 5′-mivaRNAI(A-23) and 3′-mivaRNAI-138 [22]. Other studies have shown that the HAdV-5 VA RNAI is cleaved into two prominent mature 3’-mivaRNAs: 3′-mivaRNAI-138 and 3′-mivaRNAI-137, which differ in the first 5’ nucleotide, mapping either to the position 137 or 138 on VA RNAI [19,21,88].

Elimination of the Dicer protein by siRNA or shRNA approaches enhanced accumulation of full-length VA RNA, inhibited eIF2α phosphorylation and elevated virus replication whereas transient overexpression of Dicer results in an inhibition of HAdV-5 replication [86]. Taken together, these results indicate that Dicer may function as an anti-viral protein by executing its negative effect on virus growth via VA RNA cleavage. In a simplistic extension of this model, the apical stem and central domain (AC-CD) Dicer cleavage product should not exhibit a pro-viral activity in vivo. However, in vitro studies indicate that the AC-CD Dicer cleavage product still retains some inhibitory activity on PKR as well as a suppressing effect on OAS1, hence supporting a pro-viral role (see also Section 3.3.2.) [12]. Another study showed that elimination of Dicer did not have a detectable effect on HAdV-5 late protein expression in HEK293 cells, thereby challenging the proposed anti-viral status of Dicer [89]. These discrepancies are probably due to the usage and comparison of different in vitro and in vivo experimental systems. Thus, additional studies are necessitated to understand whether the proposed Dicer anti-viral activity is virus type (i.e., HAdV-5) or cell specific and whether the Dicer VA RNA cleavage product AC-CD is detectable at all in different virus infections.

For miRNAs to function, they need to associate with the Argonaute (Ago) family of proteins, to form a functional miRNA-induced silencing complexes (miRISC). Only one of the two miRNA strands, either “5p” or “3p”, is loaded into miRISC, which implies that miRNA duplexes undergo a rigorous strand selection procedure [90]. Similarly, to cellular miRNAs, the mivaRNAs associate with the Ago proteins, thereby forming a functional miRISC in virus-infected cells [21,22,86,89]. Initial, low-throughput sequencing experiments, showed that approximately 80% of Ago2-containing miRISC in the HAdV-5-infected HEK293 cells were associated with mivaRNAs [88]. Interestingly, VA RNAII, which usually is expressed about 20-fold less compared to VA RNAI, accounted for more than 50% of the small RNAs in miRISC in the same study [88]. High-throughput RNA sequencing experiments have shown that less, 15% of small RNA reads in the immunopurified miRISC corresponded to the mivaRNAs in the HAdV-5-infected A549 cells [21]. However, similarly to the original finding by Xu and co-workers [88], the mivaRNAII is the main species in the miRISC [21].

Assembly of mivaRNA into miRISC is highly asymmetric with the 3’ strand of both HAdV-5 VA RNAs incorporated with higher efficiency compared to the 5’-strand [21,35]. The mivaRNA strands are named as follows: the “5p” strand is labelled as 5’-mivaRNA, whereas the “3p” strand is known as 3’-mivaRNA (Figure 1) [88]. Asymmetric mivaRNA incorporation has also been seen for other HAdV types. In HAdV-4, HAdV-5, and HAdV-11, the 3’-mivaRNAI is the favored strand for miRISC assembly. In contrast, the 5’-mivaRNAII strand is preferentially assembled into miRISC in the HAdV-37-infected cells [22]. The sequence analysis also demonstrated that the 3´-mivaRNAI are heterogeneous at their 5´-terminus, probably resulting from a slight variation in Dicer cleavage [22]. Even though, HAdV-5 3’-mivaRNAI is the preferred strand for miRISC assembly, the complexes generated are unstable with a low target mRNA cleavage activity compared to 5’-strand of mivaRNAI [35]. Despite the low target mRNA cleavage, other studies have shown that 3’-mivaRNAs can efficiently block translation of a reporter gene mRNA containing mivaRNA complementary sequence element [19,21].

The 5’-mivaRNAI, which accounts for a minor fraction of the mivaRNAs in miRISC, seems to form stable miRISC complexes that are highly active in guiding target mRNA cleavage [21,88]. However, the 5‘-mivaRNAI silence reporter genes less potently than 3’-mivaRNAs, agreeing with the observation that 3′-mivaRNAs are more efficiently incorporated into miRISC [88]. Since VA RNAI is transcribed by the RNAPIII, approximately 50% of the VA RNAI have a 5’-triphosphorylated end [34]. Curiously, the Ago2-bound 5’-mivaRNAI was shown to contain mainly a 5′-monophosphate end [35]. This dephosphorylation step is carried out by the cellular dual specificity phosphatase 11 (DUSP11) [91]. Depletion of DUSP11 or expression of its catalytic mutant reduces 5’-mivaRNAI but not the 3’-mivaRNAI accumulation. Further, the 5’-mivaRNAI is less efficiently bound to the Ago1 and Ago2 proteins in DUSP11 knockout cells compared to wild type cells. Hence, 5’-mivaRNAI dephosphorylation by the DUSP11 protein regulates 5’-mivaRNAI accumulation and its incorporation into active miRISC [91].

It should be remembered that VA RNAI transcription initiates at two start sites generating the major VA RNAI(G), and the minor VA RNAI(A) species [34]. Dicer cleavage of VA RNAI(A) results in production of a 5´-mivaRNAI(A), which is 3 nucleotides longer than the mivaRNAI(G) processed from the VA RNAI(G) transcript (Figure 1). This 3-nucleotide difference might be important, as the 5´-mivaRNAI(A) generates active miRISC complexes whereas the 5’-mivaRNAI(G) appears to generate nonfunctional complexes [35]. The preferred association of the 5’-mivaRNAI(A) with the miRISC seems to be a common feature for at least three HAdV types (HAdV-4, HAdV-5, HAdV-11) [22].

All the described studies have been carried out during lytic HAdV infections. However, HAdV-5 can also induce long-term persistent infections [8]. Similarly, to the lytic infections, mivaRNAs are also detected in miRISC in persistently infected B cell line BJAB, with mivaRNAII showing higher level of accumulation compared to mivaRNAI [92]. MivaRNAs are also detected, although at a very low level, in tonsillar T lymphocytes derived from patients diagnosed with tonsillar diseases and tested positive for HAdV-2 and HAdV-5 infections [93].

Considering the enormous accumulation of mivaRNAs in HAdV-infected cell, it seems reasonable that this would affect cellular miRNA incorporation into miRISC. Indeed, cellular miRNA association with Ago2 containing miRISC was reduced approximately 5-fold in HAdV-5-infected cells [88]. Particularly, incorporation of the cellular hsa-let-7 family members into miRISC was affected in HAdV-5-infected A549 cells [21]. It has been hypothesized that the mivaRNAs may function as a sponge to sequester Ago2, which would have effects on the cellular miRNA presence and quantity in miRISC. A reduction of cellular miRNAs in miRISC, which potentially may have an antiviral activity, can therefore be one of the reasons why miRISC efficiency is reduced in HAdV-5-infected as well as in cells overexpressing VA RNAI [18,84].

### 4.2. Function

Although the details of mivaRNA biogenesis are fairly well-established, there is still a limited understanding about the mivaRNA functions in the virus-infected cell. To test if the mivaRNAs are needed for a productive lytic infection of HAdV-5, the 5’-mivaRNAI and 3’-mivaRNAI seed sequences were mutated in the VA RNAI gene in the HAdV-5 virus genome. Surprisingly, the mivaRNA mutant viruses were not defective in PKR inhibition, mivaRNA processing nor virus late protein production. Two independent studies have suggested that the HAdV-5 VA RNAI-derived mivaRNAs are not essential for lytic virus growth, at least not in tissue culture cells [86,89]. However, it is possible that mutations in the mivaRNAII seed sequences will have a more profound effect on virus replication compared to the mivaRNAI (see below).

**Figure 3:**
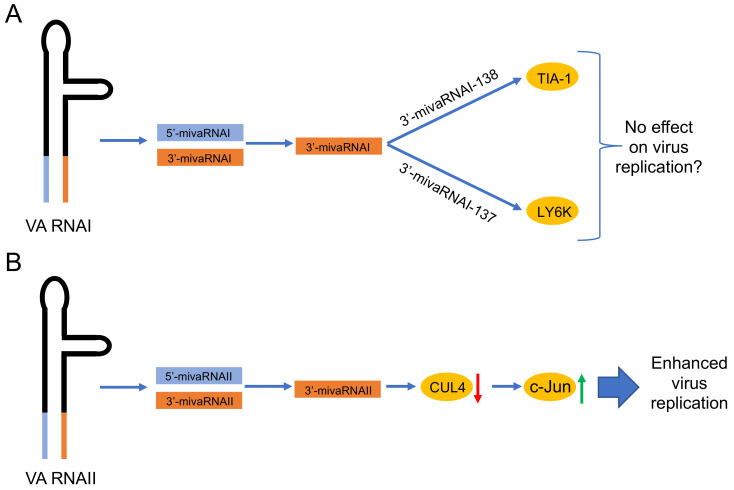
Proposed functions of the HAdV-5 mivaRNAs. (**A**) Validated targets (indicated in the yellow circles) of the mature 3’-mivaRNAI [19,21]. Since mutations in the 5’-mivaRNAI and 3’-mivaRNA sequences do not affect virus growth [86,89], the possibility that these mivaRNAs do not have an effect on virus growth is mentioned. (**B**) Proposed function of the 3’-mivaRNAII, which reduces CUL4 expression [94]. Low levels of the CUL4 protein will stabilize the c-Jun protein, which in turn enhances HAdV-5 growth. Red arrow, downregulation; green arrow, upregulation.

Even if the mutational studies have not confirmed the essential role of mivaRNAI in virus infection, other experimental approaches have shown that mivaRNAs are functional and they may have a role in virus-infected cells (Figure 3). For example, the mivaRNAs can inhibit reporter gene expression when engineered to target complementary sequences present in a 3´-UTR [18,19,20,21,87]. Further, blockage of the mivaRNAs with the antagomirs reduced HAdV-5 production [20]. Since a fraction of the mivaRNAIIs were detected in polyribosomes, it suggested that mivaRNAs may interfere with the translation efficiency of some of the mRNAs [88]. Together, these results still indicated a potential role of the mivaRNAs in regulating translation of cellular and/or viral mRNAs.

Based on miRNA target predictions, the HAdV-5 genome appears to have been selected against having highly complementary mivaRNA target sequences within the viral mRNAs [19,88]. Since the cellular transcriptome is much more complex compared to the HAdV-5 transcriptome, it is reasonable to assume that the mivaRNAs may target cellular mRNAs. A pioneering cDNA microarray study by Aparicio and co-workers identified 637 upregulated and 462 downregulated mRNAs in VA RNA expressing cells. In the following analyses, it was shown that the best cellular target mRNAs contained complementary sequence to the 3’-mivaRNAI-138. One of the identified mivaRNAI-138 targets was a splicing factor T-cell intracellular antigen (TIA-1) mRNA, with mivaRNAI-138 complementarity sequence in the 3´-UTR. The group further showed that TIA-1 expression was reduced at the mRNA and protein levels in virus-infected cells expressing functional mivaRNAs [19]. Since alternative RNA splicing controls HAdV lifecycle [95], TIA-1 protein downregulation by mivaRNAI-138 could contribute to the temporal shift in HAdV RNA splicing [96]. However, at present, it is not clear whether this regulation of TIA-1 expression has any significant beneficial effects on HAdV growth 

Analysis of mRNAs enriched in miRISC in HAdV-5-infected cells has revealed additional mRNA targets which, have complementarity to mivaRNAs [21]. Among different mivaRNAI species, the 3’-mivaRNAI-137 was shown to be the most abundant in the miRISC and was therefore chosen to identify cellular mRNA targets. High stringency analysis identified 20 candidate genes encoding proteins involved in apoptosis (e.g., BOK), mitochondrial processes (e.g., BRP44), membrane-associated processes (e.g., SMAGP, TMEM222) or cell growth (e.g., LY6K) regulation. Most of these 20 genes were also down-regulated after mivaRNAI mimic transfection with preference for the genes containing the mivaRNAI target sites in the coding region or 3’ UTR regions. Even though the previously identified TIA-1 [19] was not among the targets in this study [21], another gene, lymphocyte antigen 6 family member K (LY6K), emerged as a target identified by the both studies. Since the 3’-mivaRNAI-137 and 3’-mivaRNAI-138 seed sequence is shifted by only one nucleotide, it is likely that they both can target LY6K mRNA. However, it is still unclear what is the functional outcome of LY6K down-regulation during HAdV infection.

Similarly to the mivaRNAI, also the mivaRNAII species show enrichment in the miRISC in HAdV-5-infected cells [21,22]. However, the incorporation rate of mivaRNAII seems to be virus type specific. Northern blot analyses have shown that the HAdV-37 mivaRNAII associates significantly better with the Ago2-based miRISC compared to the HAdV-5 and HAdV-4 mivaRNAII [22]. Notably, *in silico* bioinformatics predictions have indicated that mivaRNAII from different virus types (i.e., HAdV-4, HAdV-5, HAdV-37) may target the same cellular genes [22]. Despite of that, a direct experimental evidence that mivaRNAII can indeed target cellular mRNAs has been missing for a long time. However, a recent study has shed a light on this topic, by showing that HAdV-5 most abundant mivaRNAII, 3’-mivaRNAII-138, actually has a defined mRNA targets in cells [94]. Transient overexpression of the 3’-mivaRNAII-138 mimic identified 8 cellular genes, which were clearly down-regulated. Notably, siRNA knockdown of one of them, CUL4 (cullin 4A), significantly increased HAdV-5 replication. Mechanistically, CUL4 mRNA down-regulation by 3’-mivaRNAII was further connected to the accumulation of a transcription factor c-Jun and activation of the Jun-N-terminal kinase (JNK) signaling cascade (Figure 3). Activation of the JNK signaling was further proposed to cause enhanced HAdV-5 replication, although the exact details still remain to be resolved [94].

## 5. MLP-TSS-sRNA

### Synthesis and Structure

**Figure 4:**
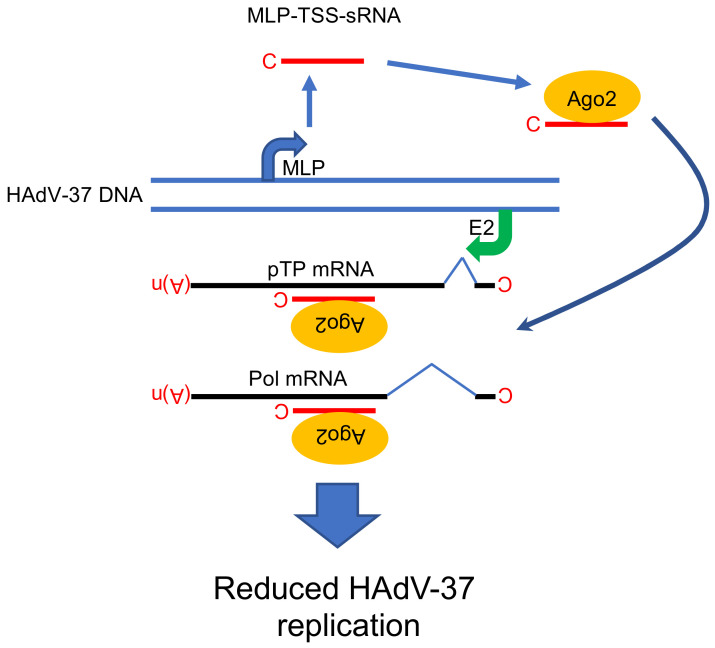
Proposed functional role of MLP-TSS-sRNA in HAdV-37 cells. The MLP-TSS-sRNA is in the complex with the Ago2 protein and anneals to its complementary sequence on two viral mRNAs (pTP and Pol). Abbreviations: C; (m7G)-cap structure, MLP; Major Late Promoter, E2; E2 promoter, pTP; preterminal protein, Pol; virus DNA polymerase, (A)n; poly(A) tail, Ago2; Argonaute 2 protein, MLP-TSS-sRNA; Major Late Promoter-Transcription Start Site-small RNA. Model is based on the study [16].

Based on the HAdV-2 and HAdV-5 studies, it was believed that the mivaRNAs were the only functional viral small RNAs generated during a HAdV infection [17,21]. This viewpoint has been recently challenged by a study showing that an unusual, 31 nucleotide long, capped sncRNA accumulates to high levels in HAdV-37-infected cells [16]. Since this sncRNA originates from the viral major late promoter (MLP) transcriptional start site (TSS), it has been named as the MLP-TSS-sRNA (Figure 1 and Figure 4). In addition to HAdV-37, the same MLP-TSS-sRNAs can be also detected at low levels in other virus type (HAdV-4, HAdV-5, HAdV-11) infected cells, although it shows the highest accumulation in the HAdV-37-infected cells.

Mechanistically, the MLP-TSS-sRNA is produced by promoter proximal stalling/termination of RNA polymerase II (RNAPII) transcription, which explains the presence of a (m7G)-cap structure at the 5’ end of the MLP-TSS-sRNA. The MLP-TSS-sRNA is highly stable in cells and is efficiently incorporated into Ago2-containing functionally active miRISC complexes in the HAdV-37 infected cells. Even though, MLP-TSS-sRNA is found in miRISC, it does not require the Dicer protein for its processing or the Exp5 protein for its nuclear export. Similarly, to the mivaRNAs, MLP-TSS-sRNA containing miRISC can inhibit expression of reporter genes with complementary target sequences. Because the MLP-TSS-sRNA is transcribed from the opposite strand to the HAdV-37 DNA polymerase (Pol) and preterminal protein (pTP) mRNAs, it was shown to act in *trans* to reduce Pol and pTP mRNA expression. Consequently, the MLP-TSS-sRNA has a negative effect on virus DNA replication, something the authors speculated could have an importance in establishment of persistent HAdV infections. Hence, the MLP-TSS-sRNA covers at least three unique features: It is produced as a single-stranded, (m7G)-cap-modified molecule, it is very stable, due to the small stem-loop structure at its 3’ end (Figure 1); and it forms functional Ago2-miRISC able to repress complementary targets (Figure 4).

Taken together, in contrast to the mivaRNAs, the MLP-TSS-sRNA is generated by a non-canonical miRNA pathway, it inhibits virus growth and may have a more general function as a regulator of viral DNA replication since it is detectable in all serotypes tested (Figure 4) [16].

## 6. Conclusions and Future Perspectives

The last 60 years of research have expanded our understanding about the VA RNA regarding its synthesis, structure and function. However, there are still a number of interesting unanswered questions which need to be addressed. For example, a co-crystallization of VA RNAI and PKR is desirable to understand the details how VA RNAI binds to and inhibits PKR function. Moreover, since inflammation is such a central part of a virus infection, the recent finding that VA RNAI blocks NLRP3 inflammasome activation [63], urges for more detailed studies about the anti-inflammatory role of VA RNAI and potentially of VA RNAII.

Limited data is available about the mivaRNA function during HAdV infection. Current knowledge about the VA RNA and mivaRNA functions are restricted to the studies based on HAdV-5 infections. Thus, it is important to expand the functional studies also to mivaRNAs expressed from other HAdV types, including more pathogenic and re-emerging HAdV-7 and HAdV-55 [29,97]. Further, it is well established that some HAdV types cause persistent infections in human adenoids and tonsils [9]. Even though mivaRNAs are detected in these infections, we do not know their function or potential cellular targets [92,93]. Considering that HAdV-5 mivaRNAI is not needed for lytic virus growth [86,89], it is possible that mivaRNAI may serve a primary function during the establishment and/or maintenance of long lasting infections in particular cell types. Hence, usage of persistent infection mimicking cell models and patient-derived material may unravel the mivaRNA roles in these enigmatic infections.

The discovery of the non-canonical MLP-TSS-sRNA also solicits for further studies. Do the other viral promoters also produce Ago2-associated TSS-sRNAs? Are the Ago2-associated TSS-sRNAs unique to a HAdV infection or do they also exist in other virus infections? It is known that mouse embryonic stem cells produce functional TSS-miRNAs [98]. It will be of interest to study whether HAdV infections can control accumulation of the similar TSS-miRNAs in human cell lines and tissues. Finally, considering the unique structural and functional features of the MLP-TSS-sRNA, it will be attractive to test if the modified MLP-TSS-sRNA can be used to target cellular mRNAs for therapeutic purposes.

Taken together, we believe that the coming years will presents us with several exiting possibilities to explore the structural and functional details of the VA RNA, mivaRNA and TSS-sRNA, with an ultimate aim to apply this gained knowledge to design advanced HAdV vectors. These studies are especially important since modified adenoviruses have turned out to be suitable delivery vectors to vaccinate against SARS-CoV2 infections [5,6].
